# Cespitulones A and B, Cytotoxic Diterpenoids of a New Structure Class from the Soft Coral *Cespitularia taeniata*

**DOI:** 10.3390/md12063477

**Published:** 2014-06-05

**Authors:** Yu-Chi Lin, Shih-Sheng Wang, Chung-Hsiung Chen, Yao-Haur Kuo, Ya-Ching Shen

**Affiliations:** 1 School of Pharmacy, College of Medicine, National Taiwan University, Taipei 100, Taiwan; E-Mails: z10108042@email.ncku.edu.tw (Y.-C.L.); sionchen@ntu.edu.tw (C.-H.C.); 2 Department of Life Sciences, National Cheng Kung University, No. 1 University Road, Tainan 701, Taiwan; 3 Department of Marine Biotechnology and Resources, National Sun Yat-Sen University, Kaohsiung 804, Taiwan; E-Mail: aska@newbellus.com.tw; 4 Division of Herbal Drugs and Natural Products, National Research Institute of Chinese Medicine, Taipei 112, Taiwan; E-Mail: kuoyh@nricm.edu.tw

**Keywords:** *Cespitularia taeniata*, diterpenoid, cespitulone, cytotoxicity

## Abstract

Two novel diterpenoids, cespitulones A (**1**) and B (**2**), were isolated from extracts of the soft coral *Cespitularia taeniata*. Both compounds possess an unprecedented bicyclo [10.3.1] ring system with C-C bond connections between C-10 and C-20, and between C-20 and C-11. Their structures were elucidated on the basis of extensive spectroscopic analyses. Compound **1** exhibited significant cytotoxicity against human medulloblastoma and colon adenocarcinoma cancer cells.

## 1. Introduction

**Figure 1:**
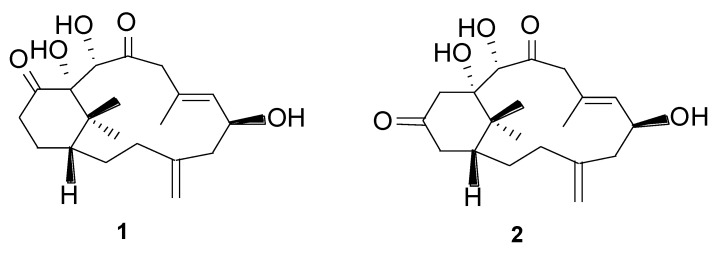
Cespitulones A (**1**) and B (**2**) isolated from soft coral *Cespitularia taeniata*.

Marine soft corals are an excellent source of secondary metabolites with novel structures and interesting biological functions [1,2,3,4,5]. Members of the genus *Cespitularia*, along with morphologically similar *Xenia* species, inhabit the coral reefs along the coasts of Taiwan. These interesting cnidarians have brilliant colors and their outer layers are covered with thick mucilage. Previously, several members of the genus *Cespitularia* were reported to contain a series of complex terpenoids, including cespitularins, nitrogen-containing diterpenoids, cespihypotins, cespitulins and cespitulactones [6,7,8,9,10,11,12]. These diterpenoids are thought to be derived from geranylgeranyl pyrophosphate and 1S-verticillene, involving interesting biogenetic pathways similar to those that generate the taxane diterpenes [13,14,15]. To explore novel bioactive metabolites from these invertebrates, we continued our study on *Cespitularia taeniata*, and have now isolated two novel diterpenoids, designated as cespitulones A (**1**) and B (**2**) (Figure 1). Both compounds possess an unprecedented bicyclo [10.3.1] skeleton. Here we report the isolation, structural elucidation, plausible biogenetic pathway, and the cytotoxicity of **1** and **2**.

## 2. Results

**Figure 2:**
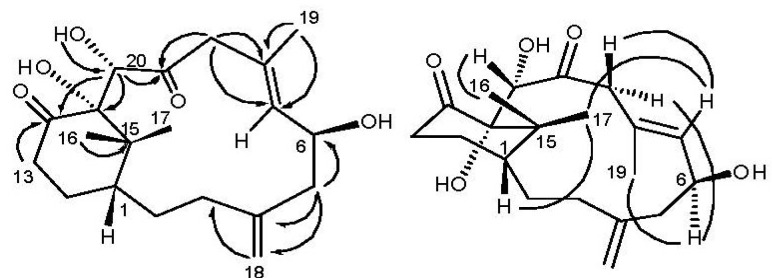
Selective HMBC (hook) and NOESY (curve) correlations of **1**.

Cespitulone A (**1**) was obtained as an amorphous solid that analyzed by HRESIMS for the molecular formula C_20_H_30_O_5_, having six degrees of unsaturation. The presences of hydroxyl and carbonyl functions were indicated by IR absorptions at 3419 and 1703 cm^−1^. The ^1^H- and ^13^C NMR spectra (Table 1), along with DEPT NMR data, confirmed the presence of two carbonyls (δ_C_ 212.4 and 207.4), and illustrated a trisubstituted olefin (δ_C_ 131.7 CH, 133.6 C; δ_H_ 5.58 d, *J* = 9.1 Hz), a 1,1-disubstituted olefin (δ_C_ 144.9) with an exomethylene group (δ_C_ 115.5 CH_2_; δ_H_ 4.87s, 4.92s), and one aliphatic quaternary carbon (δ_C_ 45.6, C-15). In addition, two oxygenated methine carbons (δ_C_ 69.8 CH, 77.4 CH), an oxygenated tertiary carbon 34.3 and δ_C_ 24.6), and three methyl groups (δ_C_ 26.9, 27.2, (δ_C_ 89.1 C), six methylene carbons (δ_C_ 31.5, 38.7, 46.9, 47.7, 18.6) with their corresponding proton chemical shifts (δ_H_ 1.54, 1.32, 1.88) were observed. Since **1** contained two carbonyls and two double bonds, the carbon framework of cespitulone A must be bicyclic. Analysis of the COSY NMR data for **1** established the connectivities of H-9/H-9, Me-19/H-7/H-6, H-6/H-5/H-18/H-3/H-2/H-1 and H-13/H-14/H-1. These coupled with the HMBC NMR correlations of H-20/C-10, C-11, C-12, and H-9/C-10, and H-13/C-12, allowed the positions of the carbonyls at C-10 and C-12 and the hydroxyl at C-20 to be assigned. Thus, C-20 could be positioned between the C-10 carbonyl and the tertiary oxygenated C-11 carbon. This suggested that **1** contains an unusual bicyclic system in which the C-20 methyl group (as in cespitularines) was somehow modified and incorporated into the ring system. Analysis of other HMBC correlations, including Me-16/C-11, C-15; Me-17/C-11, C-15; H-9/C-7, C-8 as well as H-5/C-4, C-6, C-18, allowed the proposed bicyclo [10.3.1] ring system to be assigned (Figure 2). The relative configuration of **1** was determined by analysis of NOESY NMR data based upon the assumption that **1** has the same absolute C-1 (H-1β) configuration as that of the *Cespitularia*-derived cespitulactams, cespitularines, cespihypotins and toxoids [16,17].

**Table 1:** ^1^H and ^13^C NMR data for **1** ^a^.

No	δ_H_ (mult, *J*, Hz) ^b^	δ_C_^c^	HMBC	COSY
			^1^H-^13^C	^1^H-^1^H
1	1.54 (m)	45.3	11, 15, 16	2, 14
2	1.10 (m), 1.46 (m)	31.5	1	1, 3
3	2.00 (m), 2.23 (m)	38.7		2, 18
4		144.9		
5	2.14 (m), 2.68 (m)	46.9	4, 6, 18	6, 18
6	4.52 (td, 9.0, 5.5)	69.8	4, 5, 7	7
7	5.58 (d, 9.0)	131.7		6, 19
8		133.6		
9α	2.63 (d, 14.0)	47.7	6, 7, 8, 10	9b
9β	4.00 (d, 14.0)		7, 8, 10, 19	9a
10		212.4		
11		89.1		
12		207.4		
13	2.77 (m), 2.53 (m)	34.3	12	14
14	2.00 (m), 1.60 (m)	24.6		1, 13
15		45.6		
16	1.54 (s)	26.9	1, 11, 15, 17	
17	1.32 (s)	27.2	1, 11, 15, 16	
18	4.87 (s), 4.92 (s)	115.5	3, 5	3, 5
19	1.88 (s)	18.6	7, 8, 9	7
20	4.14 (d, 3.0)	77.4	10, 11, 12, 15	OH
20-OH	4.30 (d, 3.0)			11, 20

^a^ Data were recorded in CDCl_3_ on 500 MHz; chemical shifts (δ) and coupling constants are given in ppm and Hz, respectively; ^b^ Assignments made by COSY and HMQC; ^c^ Assignments made by HMQC and HMBC; Multiplicities determined by DEPT.

**Figure 3:**
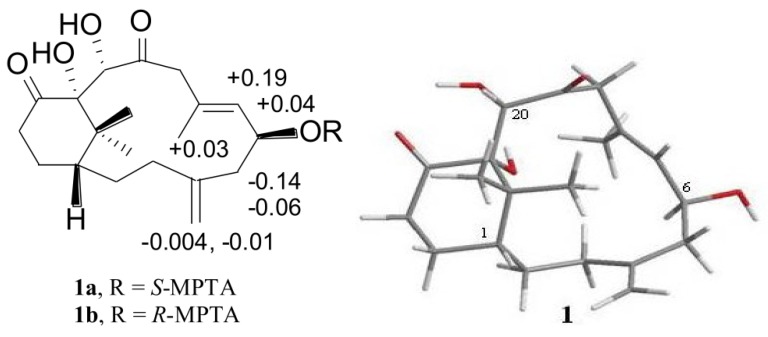
Mosher’s reaction products **1a** and **1b**, which show δ*_S_*–δ*_R_* values (ppm); Computer-generated perspective models for **1** using MM2 force field calculation.

The presence of NOESY correlations among H-1/Me-16/Me-17, H-20/Me-16, H-7/Me-17 agreed with β-configuration of Me-16, Me-17 and H-20, while H-6 was assigned an α-configuration on the base of correlations of H-6/Me-19/H-9α and H-7/H-9β (Figure 2). The configuration of the hydroxyl at C-6 was further determined by Mosher’s reactions to yield compounds **1a** and **1b ** [18]. The results, illustrated in Figure 3, suggested that the C-6 has the *S* configuration. A computer-generated perspective structure for **1** is shown in Figure 3 by CS Chem 3D version 9.0 using MM2 force field calculation for energy minimization. The results also suggested that C-6 has S configuration and C-11 hydroxy group is α-oriented.

**Figure 4:**
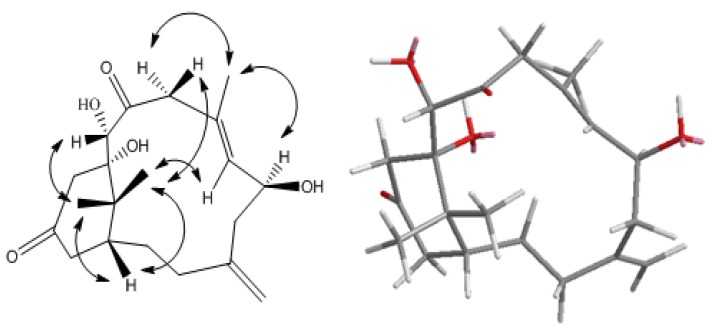
Key NOESY correlations and computer-generated perspective model for **2** using MM2 force field calculation.

Cespitulone B (**2**) was isolated as a colorless amorphous solid. The molecular formula, C_20_H_30_O_5_ (∆ = 6), was determined by HRESIMS with a pseudomolecular ion at *m/z* 373.1993 [M + Na]^+^, indicating that it is an isomer of **1**. Analysis of IR bands revealed the presence of hydroxyl (3419 cm^−1^) and carbonyl (1700 cm^−1^) functions. Comparisons of the ^1^H- and ^13^C NMR (Table 2) and DEPT data with those of **1** indicated similar functionalities of both compounds. Analysis of COSY and HMBC NMR correlations also revealed similar arrangement of each functional group around the 13-membered ring, including a 1,1-disubstituted olefin (δ_C_ 144.7) with an exomethylene group (δ_C_ 115.5 CH_2_; δ_H_ 4.87 s, 4.95s), a C-6 oxygenated methine carbon (δ_C_ 70.9 CH; δ_H_ 4.57 td, *J* = 9.6, 5.7 Hz), a trisubstituted olefin (δ_C_ 132.1 CH, 135.3 C; δ_H_ 5.54 d, *J* = 9.6 Hz), a C-10 carbonyl carbon (δ_C_ 213.9), a C-20 oxygenated methine carbon (δ_C_ 79.3 CH; δ_H_ 4.46 d, *J* = 3.0 Hz), an oxygenated tertiary carbon (δ_C_ 81.6 C), and the C-15 quaternary carbon (δ_C_ 49.6) with two attached methyl carbons (δ_C_ 24.0 CH3, 27.5 CH3). The positions of two carbonyls at C-10 (δ_C_ 213.9) and C-13 (δ_C_ 212.9) and two hydroxyl groups at C-20 and C-11 were assigned on the basis of HMBC correlations (H-9/C-10, H-20/C-10, C-11, H-12/C-11,C-13, H-14/C-13, Me-16/C-11, and Me-17/C-11). Thus the only difference revealed in comparison with **1** was the location of the C-13 carbonyl group. Analysis of NOESY correlation data [H-1/Me-16, Me-17, H-20/Me-16, and H-7/Me-17 (Figure 4)], indicated the β-orientation of Me-16, Me-17 and H-20, while H-6 was assigned as α-oriented based upon correlations observed from H-6/Me-19/H-9α and H-7/H-9β. A computer-generated perspective structure for **2** is shown in Figure 4. The results also suggested that C-6 has S configuration and the hydroxyl at C-11 is β-oriented.

**Table 2:** ^1^H and ^13^C NMR data for **2** ^a^.

No	δ_H_ (mult, *J*, Hz) ^b^	δ_C_	HMBC	COSY
			^1^H-^13^C	^1^H-^1^H
1	1.32 (m)	47.6	11,15, 16	2, 14
2	1.88, 1.93 (m)	34.3	1	1, 3
3	1.62, 2.26 (m)	40.8		2, 18
4		144.7		
5	2.07 (m), 2.81 (m)	47.2	4, 6, 18	6, 18
6	4.57 (td, 9.6, 5.7)	70.9	4, 5, 7	7
7	5.54 (d, 9.6)	132.1		6, 19
8		135.3		
9α	2.86 (d, 14.0)	48.9	7, 8, 10, 19	
9β	4.06 (d, 14.0)			
10		213.9		
11		81.6		
12	2.43 (m), 3.33 (m)	35.6	11, 13	
13		212.9	12	
14	1.67 (m), 1.87 (m)	28.2	13	1
15		49.6		
16	0.77 (s)	24	1, 11, 15, 17	
17	1.43 (s)	27.5	1, 11, 15, 16	
18	4.87 (s), 4.95 (s)	115.5	3, 5	3, 5
19	1.99 (s)	18.8	7, 8, 9	7
20	4.46 (d, 3.0)	79.3	10, 11, 15	

^a^ Data were recorded in CDCl_3_ on 500 MHz ; chemical shifts (δ) and coupling constants are given in ppm and Hz, respectively; ^b^ Assignments made by COSY and HMQC.

**Scheme 1:**
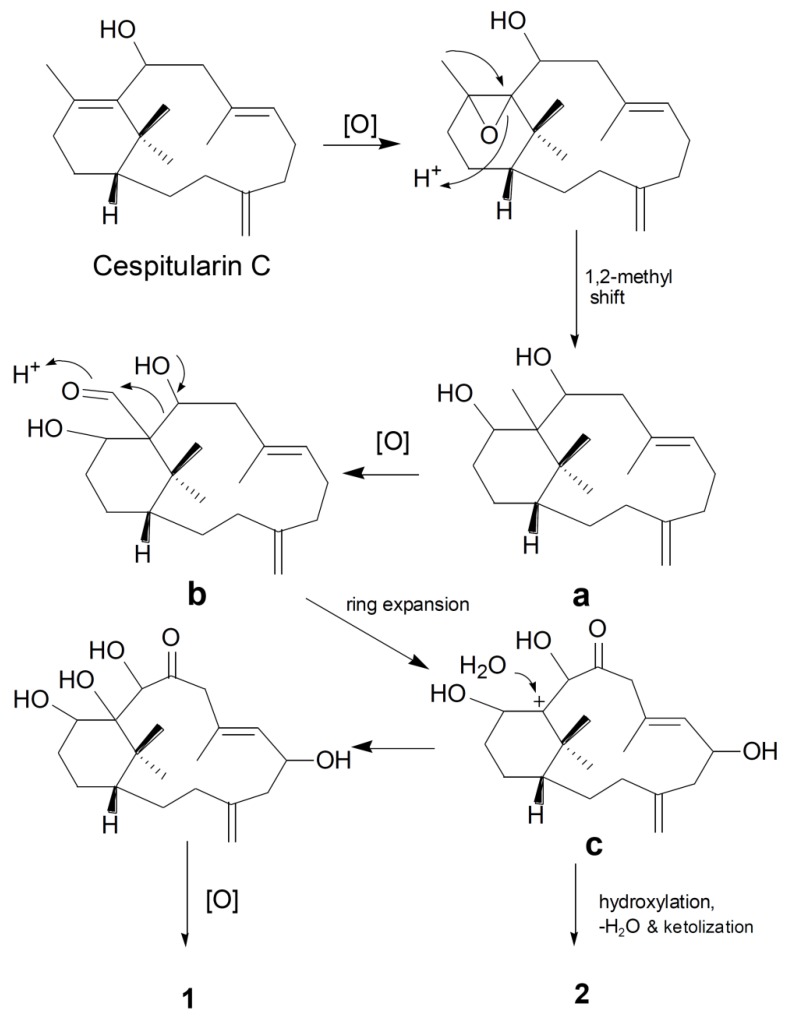
Plausible biogenetic pathway of **1** and **2**.

Scheme 1 illustrates a plausible biogenetic pathway for **1** and **2** based upon previous publications [7,8,16]. Cespitularin C might be a putative precursor of **1** and **2**. Compound **1** is probably produced via intermediates a, b and c involving steps of epoxydation, rearrangement (1,2 methyl shift), oxidation, ring expansion, hydroxylation. Compound **2** may be derived from the same pathway, but through further hydroxylation, dehydration and ketolization of cation c. The Meinwald type rearrangement may be involved to give a ketone directly in the second step [19].

The isolated compounds **1** and **2** were evaluated for cytotoxicity against human medulloblastoma (Daoy) and colon adenocarcinoma (WiDr) cancer cell lines. As a result, cespitulone A showed significant *in vitro* cytotoxicity against human medulloblastoma (Daoy) and colon adenocarcinoma (WiDr) cancer cells with IC_50_ values of 8.7 and 6.7 µM, respectively [20]. Mitomycin was used as a positive control with IC_50_ at 0.3 μM.

## 3. Experimental Section

### 3.1. General Experimental Procedures

Optical rotations were recorded on a JASCO DIP-1000 polarimeter. IR spectra were measured on Hitachi T-2001 spectrophotometer. LRESIMS and HRESIMS were taken on a JEOL JMS-HX 110 mass spectrometer. The ^1^H, ^13^C NMR, ^1^H-^1^H COSY, HMQC, HMBC and NOESY spectra were recorded on Bruker FT-300 (300 MHz for ^1^H) and a Varian UNITY INOVA 500 (500 MHz for ^1^H and 125 MHz for ^13^C) spectrometers. The chemical shifts were given in δ (ppm) and coupling constants in Hz. Silica gel 60 (Merck) was used for column chromatography. Sephadex LH-20 (Amersham Pharmacia Biotech AB, Sweden) was used for separation. LiChrospher^®^ Si 60 (5 μm, 250-10 mm, Merck, Germany) and LiChrospher^®^100 RP-18e (5 μm, 250-10 mm, Merck, Germany) were used for NP-HPLC and RP-HPLC (Hitachi), respectively.

### 3.2. Extraction and Isolation

The soft coral (1.1 Kg, freeze-dried), collected at a depth of 20 m in October 2004, was extracted with mixture of CH_2_Cl_2_/EtOH (1:1), and the crude extract was partitioned between EtOAc and H_2_O (1:1). The EtOAc-soluble fraction (100 g) was subjected to a Si gel column (*n*-hexane/EtOAc, 15:1–0:1; EtOAc/MeOH, 50:1–2:1) to give fractions 1-12. Fraction 6 (3.1 g) was chromatographed on a LH-20 Sephadex column (MeOH) and separated further by HPLC column (Si gel, *n*-hexane–CH_2_Cl_2_–MeOH, 20:20:1) to furnish cespitulone A (**1**, 3 mg). Fraction 8 (1.7 g) was further separated on a Sephadex LH-20 column using CH_2_Cl_2_-MeOH(4:1) to give 5 fractions (8-1~8-5). Fraction 8-4 (779 mg) was further separated with NP-HPLC column (*n*-hexane–CH_2_Cl_2_–MeOH, 20:20:1) and with RP-HPLC column (MeOH–H_2_O–CH_3_CN, 65:30:5) to afford **2 ** (9 mg).

### 3.3. Spectroscopic Data

*Cespitulone A* (**1**): amorphous solid, [α]^25^_D_ −58.8 (*c* = 0.2, CH_2_Cl_2_); IR (neat) ν_max_ 3419, 2924, 1703 cm^−1^; HRESIMS *m/z* 373.1989 (C_20_H_30_O_5_Na^+^, calcd 373.1991). ^1^H-NMR and ^13^C-NMR (CDCl_3_, 500/125 MHz) see Table 1 and Table 2, respectively.

*Cespitulone B* (**2**): colorless amorphous solid; [α]^25^_D_ −63.4 (*c* 0.2, CH_2_Cl_2_); IR (neat) ν_max_ 3419, 2925, 1700, 1445, 1391, 1278 cm^−1^; HRESIMS *m*/*z* 373.1993 ([M + Na]^+^, calcd for C_20_H_30_O_5_Na^+^, 373.1991). ^1^H NMR (CDCl_3_) and ^13^C NMR (CDCl_3_) data, see Table 1 and Table 2, respectively.

Preparation of (*S*)- and (*R*)-MPTA esters (**1a** and **1b**) of **1**. To a solution of **1** (0.7 mg in 0.5 mL pyridine) was added *R*-(−)- or *S*-(+)-MPTA chloride (one drop) and the solution was allowed to stand at room temperature for 12 h. After purification using preparative LC, the ester (0.6 mg, 85% yield) was analyzed by ^1^H NMR spectroscopic measurement, and ∆δ = δ*_S_* − δ*_R_* was calculated for **1**.

Compound **1a**: ^1^H NMR (CDCl_3_, 300 MHz) 5.698 (1H, td, *J* = 8.1, 3.0 Hz, H-6), 5.592 (1H, m, H-7), 1.255, 1.384 (6H, s, H-16, -17), 4.922 (1H, s, H-18), 5.041 (1H, s, H-18), 2.000 (3H, s, H-19). 

Compound **1b**: ^1^H NMR (CDCl_3_, 300 MHz) 5.655 (1H, td, *J* = 8.1, 3.0 Hz, H-6), 5.399 (1H, d, *J* = 8.1 Hz, H-7), 1.109, 1.486 (6H, s, H-16, -17), 4.926 (1H, s, H-18), 5.050 (1H, s, H-18), 1.975 (3H, s, H-19).

### 3.4. Cytotoxicity Assay

The cytotoxic activities of compounds against human medulloblastoma (Daoy) and colon adenocarcinoma (WiDr) cancer cell lines cells were assayed by the MTT (3-(4,5-dimethylthiazole-2-yl)-2,5-diphenyltetrazolium bromide) colorimetric assay as previously described [21]. Samples and control standard drugs were prepared at a concentration of 1, 10, 40, and 100 μg/mL. After seeding 2880 cells/well in a 96-well microplate for 3 h, 20 μL of sample or standard agent was placed in each well and incubated at 37 °C for 3 days. After removing the medium from the microplates, the cells were fixed with 10% formaldehyde in 0.9% saline for 30 min, dyed with 1% (w/v) methylene blue in 0.01 M borate-buffer (100 μL/well) for 30 min. The 96-well plate was dipped into a 0.01 M borate-buffer solution four times in order to remove the dye. Then, 100 μL/well of EtOH–0.1 M HCl (1:1) was added as a dye eluting solvent, and the absorbance was measured on a microtiter plate reader (Dynatech, MR 7000) at a wavelength of 650 nm. The ED_50_ value was defined by a comparison with the untreated cells as the concentration of test sample resulting in 50% reduction of absorbance. Mitomycin was used as a standard compound.

## 4. Conclusions

Our investigation on constituents of Taiwanese soft coral *Cespitularia taeniata* has resulted in the isolation of two novel diterpenoids (**1** and **2**), which possess an unprecedented bicyclo [10.3.1] ring system with C-C bond connections between C-10 and C-20, and between C-20 and C-11. Cespitulone A (**1**) exhibited significant cytotoxicity against human medulloblastoma (Daoy) and colon adenocarcinoma (WiDr) cancer cells.
